# Valedictory Address

**Published:** 1858-03

**Authors:** C. B. Chapman


Page(s) missing beneath the careful consideration of some of the brightest examples of moral and social excellence, in our own, as well as in foreign countries, to regulate the every-day transactions of their lives by a rigid system of rules. In fact, you can seldom recur to the history of any person who has added to the domains of knowledge, or whose labors have been of real benefit to nations, while members of that body composing its highest councillors, or who have been valuable teachers while standing in the sacred place, who have not marked out for themselves a system for labor adapted to their peculiar callings. Such was true of WASHINGTON and FRANKLIN, while we have a more exact record of this kind in the annals of JOHN QUINCY ADAMS. Few persons, it is true, are favored with the same opportunities for an extensive range of useful activity, through a long life, as he was, yet his laborious preparations, by hard study and severe discipline, were all matured with no expectation that he was destined to occupy places to which he was subsequently called by the voice of the whole nation. It is not in accordance with the purest character to seek such positions, but it is commendable so to educate, so to discipline yourselves as to be fitted for places of usefulness and honor, if, from the demands of the times you are called to occupy them. Those who acquire most are not the best satisfied with their attainments. A passage from the diary of this eminent statesman, (Mr. Adams,) when just upon the threshold of active life, finely illustrates the opinions he had formed of the laborious diligence to be practiced by young persons, of whatever ability they may naturally be endowed, who may be desirous of becoming useful citizens, and of acquiring for themselves an honorable name. The statesman thus writes in his diary: "I am not satisfied with the manner in which I employ my time. It is calculated to keep me perpetually fixed in that state of uselessness and disgraceful insignificancy which has been my lot for some years past. At an age when many of the characters who were born for the benefit of their fellowcreatures, have rendered themselves conspicuous among their cotemporaries, and founded a reputation upon which their memory remains, and will continue to the latest posterity—at that period, I find myself as obscure and as unknown to the world as the most stupid of human beings. In the walks of active life I have done nothing. Fortune, who claims to herself a large proportion of the merit which exhibits to public view the talents of professional men, at an early period of their lives, has not hitherto been peculiarly indulgent to me. But, if to my own mind I inquire, whether I should at this time be qualified to receive, or derive any benefit from an opportunity which it may be in her power to procure for me, my own mind would shrink from the investigation. My heart is not conscious of any unworthy ambition, nor of a desire to establish either *fame*, *honor* or *fortune* upon any other foundation than that of desert. But it is conscious, and the consideration is equally painful and humiliating; it is conscious, that the ambition is constant and unceasing, while the exertion to acquire the talents which ought alone to secure the rewards of ambition, are feeble, indolent, frequently interrupted, and never pursued with an ardor equivalent to its purposes. My future fortunes in life are therefore the object of my present speculations, and it may be proper for me to reflect further upon the same subject, and, if possible, to adopt some resolutions which may enable me to answer the great ends of my existence. I will first begin with establishing as a fundamental principle upon which all my subsequent pursuits and regulations are to be established, and the acquisition of at least a respectable reputation, is, (subject to the overruling power and wisdom of Providence) within my own power, and that on my part, nothing is wanting but a constant and persevering determination to tread in the steps which naturally lead to honor. And at the same time I am equally convinced, that I shall never attain the credit in the world which my nature directs me to wish, without such steady, patient, and persevering pursuit of the means adapted to the end I have in view, as has often been the subject of my speculations, but never of my practice.

> "Labor and toil stand firm before the throne,
> And guard, so Jove commands, the sacred place."

"The mode of life adopted almost universally by my cotemporaries and equals, is by no means calculated to secure the objects of my ambition. My emulation is seldom stimulated by observing the industry and application of those whom my situation in life gives me for companions.

"The pernicious and childish opinion, that extraordinary genius can not brook the slavery of plodding over the rubbish of antiquity (a cant so common among the heedless votaries of indolence) dulls the edge of all industry, and is one of the most powerful ingredients of the potion, which transforms some of the most promising young men into the beastly forms which, in sluggish indolence, feed upon the labors of others. The degenerate sentiment, I hope, will never obtain admission into my mind, and if my time should be loitered away in stupid idleness, I will be under the full conviction of my conscience, that I am basely bartering the greatest benefits with which human beings can be indulged, for the miserable gratifications which are hardly worthy of contributing to the enjoyments of the brute creation. And as I have grounded myself upon the principle that my character is, under the smiles of Heaven, to be the work of my own hands, it becomes me to determine upon what part of active or of speculative life I mean to rest my pretensions to eminence. My own situation and that of my country, equally prohibit me from seeking to derive any present expectations from a public career. My disposition is not military, and happily the warlike talents are not those which open the most pleasing or the most reputable avenue to fame.

"I have had transient thoughts of undertaking some useful literary performance, but the pursuit would militate too much at present with that of the profession upon which I am to depend, not only for my reputation, but for my subsistence.

I have, therefore, concluded that the most proper object of my present attention is that profession itself. And in acquiring the facility to discharge the duties of it in a manner suitable to my own wishes and the expectations of my friends, I find ample room for close and attentive application, for frequent and considerate observations, and for such benefits of practical experience as occasional opportunities may throw in my way."

I have drawn thus liberally from the private determinations of one who was preparing himself, as subsequent events show, though unconsciously to himself, for positions of uncommon usefulness as a statesman and embassador, on account of the example they afford; and who, when crowded to unwonted tension in the affairs of state, always found a fragment of time, each day, to indulge his favorite literary tastes. Allow me to urge you to follow out in your daily practice these sentiments, as they may be adapted to your own pursuits, which I have extracted from this diary so liberally, as presenting like a mirror the forecast of his subsequent long and active, yet always studious life. Mr. Adams, through his long and eventful life, applied himself with youthful diligence to whatever most strongly demanded his attention.

There is much healthful pleasure in the pursuits of science and literature, to which pecuniary returns are not to be compared. The Olympic games were established for the purpose of inuring those who engaged in them to hardships, to increase the energies of the physical man by use. So the mental energies should be exercised in such way as will impart strength and vigor, in their necessary use at subsequent times.

In order to this, it is well to select such studies as are designed to impart a systematic knowledge of common events, as well as of the properties of the great diversity of forms of matter that is constantly under our observation—for the study of each opens a wide field of inquiry, and furnishes a constant source of amusement—it gives free access to a fountain from whence the curious may gratify his tastes. In these pursuits the end does not always terminate in the gratification of a philosophic taste, however laudable in itself the cultivation of such taste may be. The recent observations of Lieut. Maury with regard to the winds and ocean currents have diminished the time occupied in a voyage from New York to San Francisco from 183 to 135 days, not only saving much time to the voyager, and diminished wear of his ship, but rendering it more safe and uniform, by knowing times and places in which these natural phenomena may be made to subserve the interests of men.

The height of ocean waves had long been a subject of speculation. No one had applied the rules of science, to ascertain their height—the spaces between their crest—or their velocity. This omission attracted the attention of Professor Scoresby. Following his first impulse, he took passage in the since ill-fated Arctic, at a season that best suited his purpose.

At early dawn one tempestuous morning, a man was observed in unique costume, passing, sailor-like, from wheel-house to deck, and from place to place in such mysterious way, that fears were entertained that there was a fellow-passenger who was bereft of reason; but such fears were soon known to be unfounded, as he was observed to receive well-marked respect from the officers of the ship. The whole period of a violent storm, which sometimes approached a hurricane, was thus occupied in scientific observations, while others could only regard their safety in the cabin. As a result of this adventure, we are furnished with a tolerably reliable description of the *altitude*, *breadth*, and *velocity* of ocean waves. A scientific observer has thus recorded his speculations: "The vast sea-weed meadows of the Atlantic, which cover a space nearly seven times larger than France, teem with life; and deep sea soundings, which reveal the ocean floor at the greatest depths, tell us that the bottom of the ocean is in many places paved with calcareous silicious shells. The study of those sunless treasures, which are now recovered with much ingenuity by Brooks' deep-sea sounding lead, suggests new views with regard to the physical economy of the ocean, inasmuch as they are atoms of which mountains are formed. The ocean bed is full of irregularities, and we may easily imagine that, as the steamer pushes her way across the vast Atlantic, that although her water-path was trackless, we are yet hastening from mountain to mountain, across or along valleys, over table-lands, and in fact all the irregularities of the ocean floor. These soundings reveal to us that the Atlantic basin is a vast trough, bounded on one side by America, and on the other side by Africa, and that rising out of this trough are mountains, higher than the loftiest snow-crowned Himalayas, from peak to peak of which huge whales hold their course with the same precision with which eagles fly from crag to crag in their mountain-home; and valleys deeper than any trodden by the foot of man, within whose oozy folds the great waters lie in perpetual repose."

We may turn to facts which reveal the natural destiny of nations, in their economic and social condition, for we find an increasing disproportion in the animal and vegetable productions of the Old and New World, for civilization seems to augment her demands upon the produce of the ground.

| | | |
|---|---|---|
| Europe has 89 | inhabitants to | square mile. |
| Asia.........32 | do. | do. |
| Africa .......14 | do. | do. |
| America..... 4 | do. | do. |

These examples are given, not for the novelty which they in themselves possess, so much as to show the value of a well regulated appreciation of science, and in what variety of ways it may be made to subserve the interests and social comfort of nations. Let us watch with jealous care the pretensions of every effusion which is put forth under the name of science. Let us always assure ourselves that such are founded upon the principles of eternal truth—than which there is nothing in the universe more beautiful.

The motives which have impelled votaries to different departments of science have been as various as those pursuits are diverse. Humboldt declares that he was pervaded with an almost irresistible desire to become a scientific traveler, when but a youth, on seeing a mammoth palm in the garden of plants in Paris. In order to effectually prepare for the wide range of observation which he laid out for himself, it became necessary that he should master, not only a diversity of departments of science, but that he should become familiar with a number of foreign languages. Consequently, before his first excursion, he could converse, in addition to his native language, in *French*, *Spanish*, *Portuguese*, *Italian*, and *English.* The reflections which influenced Columbus to engage in the arduous undertaking of seeking a western continent were various, but the one on which his confidence in the existence of one beyond the Atlantic was principally founded, or to which special importance was atttached, was the fact that the bodies of two men had been cast upon the Island of Flores, differing essentially in physical characteristics from any known race. These are not Europeans, he soliloquized ; if not, they must have come to us from beyond the great waters. From this time all his mental and physical energies were committed to his long cherished purpose.

There are pleasures in these pursuits to the philosophic student, derived from the fact that he is constantly learning to interpret the phenomena of nature's handiwork. There is nothing really new in the world of matter. The most sublime truths were nearly the same countless ages ago, but they remained undiscovered to man, until one by one they have been added to the domains of revealed truth. An exalted source of pleasure in these pursuits is found in the wonderful and reliable uniformity which pervades them. Who, then, can tell how much they lose, who are strangers to serious meditation upon the wonders and beauties of nature ? How glorious does the God of nature shine in all his works ! Not a *tree*, or *shrub*, or *leaf*, or *petal*—not a *bird* or insect, but proclaims in glowing language, God made me. Next to the best of books which you can peruse, is the book of nature. From its pages you may constantly glean that which you need both for amusement and instruction. From this volume you may learn that which is most useful in your every-day employment, and that which is most beautiful in the world of animate and inanimate objects that surround you.

No common life is equal to a complete acquirement of all these lessons. Sir Isaac Newton is said to have declared, toward the close of his life, that he did not know how he might seem to others, but to himself he seemed, in his scientific acquirements, like a youth, standing upon the sea shore, and finding here a brighter pebble, and there a more curious shell, while a great ocean of truth lay all undiscovered before him.

The object of these suggestions and the narrative extracts which I have introduced into different parts of my discourse, bave been given in order to present examples that would lead you to use the best mental and physical energies which you possess in the best way ; such as may induce you to secure to yourselves the greatest amount of laudable gratification, and may lead you best to subserve the interests of your cotemporaries.

Slacken not, then, in the pursuit of whatever is true in principle or beautiful in practice. Contend manfully with indolence, with self-complacency which would simply decorate the mental and physical superstructure, leaving the foundations unstable and unsound. Cultivate, above all, self-knowledge, perseverance, piety. Leave nothing unsaid or undone, however great may be the sacrifice which duty prompts. Strive to plant, every day you live, at least one good deed in the garden of life. Though hidden and forgotten, it will revive. It may seem to be dead, but it will sprout again. The tender branches thereof shall not cease.

